# Patient with bipolar I disorder who presented with low blood lithium levels after receiving crushed tablets via a nasogastric tube: A case report

**DOI:** 10.3389/fpsyt.2022.1071721

**Published:** 2022-12-01

**Authors:** Ryoko Fujikawa, Kumiko Fujii, Yuji Ozeki

**Affiliations:** Department of Psychiatry, Shiga University of Medical Science, Otsu, Japan

**Keywords:** nasogastric tube, lithium, bipolar disorder, crushed form, low blood level

## Abstract

In clinical psychiatric cases, the placement of a nasogastric tube is occasionally considered. If a patient who presents with mania or other psychiatric conditions refuses to take drugs, they are administered via the nasogastric tube. The tablet is crushed, suspended, passed via the nasogastric line, and reaches the stomach directly. However, the effects of these processes on blood drug concentrations remain unclear. Herein, we report a patient with bipolar I disorder who presented with low blood lithium carbonate (Li) concentrations after receiving the drug via the nasogastric tube.

**Case:**

A 26-year-old woman developed manic symptoms with grandeur delusion. She was admitted to a psychiatric hospital three times after diagnosis. Her manic symptoms with delusion improved with Li and aripiprazole (ARP). Her condition stabilized with Li 800 mg/day and ARP 9 mg/day. After the Li dose was reduced to 600 mg/day, she maintained remission, with the blood level range of Li being 0.31 ∼ 0.42 mEq/L. After 1 year, she was admitted to our hospital due to a jaw deformity. During the perioperative period, treatment with oral Li was discontinued by the surgeons, and her manic symptoms recurred. During therapy with olanzapine 20 mg and Li 800 mg, her blood Li concentration was 0.67 mEq/L. The symptoms remained. Hence, the Li dose increased to 1,000 mg/day. However, she refused to take the medication. Thus, a nasogastric tube was used to administer medicines. Thereafter, the blood Li concentration decreased to 0.43 mEq/L, which was lesser than 800 mg/day. Each blood sample was collected approximately 18 h after the administration. Her symptoms remained. Thereafter, she agreed to take the medication, and the Li concentration reached 0.78 mEq/L. Then, the symptoms partly improved.

**Conclusion:**

After the administration of Li via the nasogastric tube, the Li concentration decreased, which was lower than expected. This phenomenon could be attributed to the fact that the medication was crushed, suspended, and administered via the nasogastric tube. Therefore, pulverizing and administering Li tablets via the nasogastric tube can be applied for the management of mania, however, caution should be observed because of the risk of fluctuations in blood Li levels, as in this case.

## Introduction

Lithium carbonate (Li) is a mood stabilizer commonly used in treating bipolar disorder and is considered the first-line treatment particularly in patients with acute mania (1). After oral Li is administered, it is almost completely absorbed in the intestine, and its blood concentration peaks in approximately 2 h. The half-life is about 15 h, and the drug is commonly eliminated via the kidneys. Pharmacologically, Li has antimanic, antidepressant, and neuroprotective effects by acting via different mechanisms during signal transduction after neurotransmitter receptor binding in the neurons (2, 3). Meanwhile, Li has a narrow effective therapeutic range in the blood. The periodic measurement of blood concentration and the evaluation of renal function are necessary in preventing poisoning. Maintaining a stable Li blood concentration is important in maintaining a clinically steady state. To preserve a stable Li blood concentration, treatment adherence is essential. Dehydration can lead to Li poisoning. Thus, it should be prevented to keep blood drug levels within the effective therapeutic range. In addition, attention should be paid to renal function and thyroid hormone levels during long-term use (4). According to the Japanese package insert of Li, no studies have used crushed Li tablets administered orally or via a nasogastric tube and examined its efficacy, safety, or pharmacokinetics. Hence, crushed administration is not recommended. To the best of our knowledge, there is no report regarding the use of crushed Li tablets. In this case, we administered crushed Li tablets via a nasogastric tube to control manic symptoms in a patient with bipolar I disorder who presented with severe manic symptoms. The patient’s blood Li concentration was lower than expected. We assumed that the blood Li concentration changed due to the dosage method used.

## Case description

A 26-year-old woman presented with a normal developmental history. She had one younger sister. After graduating from high school, she went to college, and she studied in Australia and the U.S. during high school and college. After graduating from college, she worked irregularly at a part-time job.

At the age of 24 years, the patient had grandeur delusions and speech and behavior inconsistencies. Police found her behavior on the street, and she was then hospitalized. Initially, she was diagnosed with acute transient psychosis and, finally, bipolar I disorder with delusion by several psychiatrists based on the Diagnostic and Statistical Manual of Mental Disorders, Fifth Edition. Elevated or irritable mood, pressure to keep talking, flight of ideas, and increased goal-directed activity were also observed. Her symptoms improved after treatment with olanzapine (OLZ) 20 mg/day and Li 800 mg/day. Thereafter, due to symptom recurrence, OLZ was replaced with aripiprazole (ARP) 12 mg/day. Then, she recovered well and worked as a part-time Japanese language teacher. She had been taking the medication to keep her condition stable. After the ARP dosage decreased to 9 mg/day and the Li dosage decreased to 600 mg/day, she could maintain remission. During this remission period, the range of blood level of Li was 0.31–0.42 mEq/L.

After 1 year, she underwent jaw deformity surgery at our hospital. At that time, because physical condition might be unstable due to surgery, treatment with Li was discontinued to prevent adverse effects. However, after the surgery, her manic symptoms recurred. About a week after the surgery, she had an agitated behavior (wanted to wear a diaper and sat naked on the floor) and was, thus, referred to the psychiatry department. Depressive symptoms such as tearfulness and anxiety were also noted, and the condition was believed to be associated with mixed features of bipolar disorder. Therefore, she was admitted to the psychiatric ward 3 days after the consultation.

The patient was transported to the psychiatric ward via a wheelchair. She had poor gaze congruency, and she stared at a single point. She occasionally responded to questions by nodding but showed hyperactive attentional transference, as evidenced by saying the name of a color or character she saw without answering. She sometimes made delusional grandeur statements such as “I must become the prime minister.” The blood test showed no abnormality in liver and kidney functions. Any lesions were not observed on brain computed tomography scan revealed.

**FIGURE 1:**
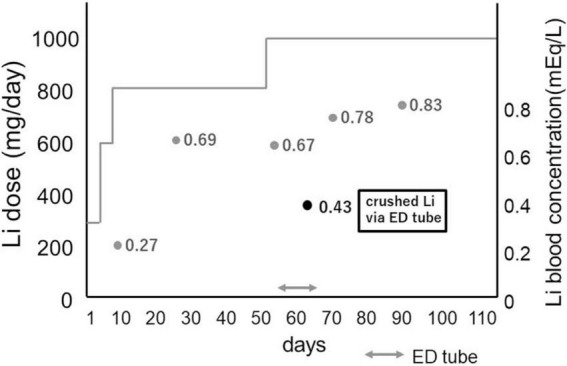
Diagram of Li dosage and progress: when Li was administered via elemental diet tube, Li blood concentration decreased compared to oral administration by the same dose. Li: lithium carbonate, ED tube: elemental diet tube.

The ARP and Li dosages were gradually increased to 24 and 600 mg/day, respectively. On the 8th day, the patient received Li 600 mg, and her blood Li concentration was 0.27 mEq/L. However, the symptoms were still severe. She repeatedly exhibited strong psychomotor agitation and inconsistent behaviors. For example, she destroyed closets. Thus, she was placed in isolation, and the Li dose was increased to 800 mg. The patient’s Li concentrations were 0.69 mEq/L on day 21 and 0.67 mEq/L on day 51. ARP 24 mg was replaced with OLZ 20 mg. During this period, the patient’s clinical condition was unstable. She continued to exhibit violent behavior. For example, she walked around naked in the protection room, yelled, singed, cried suddenly, and kicked the door angrily. Furthermore, she refused to eat or take her medications on day 51. Thus, physical restraint was applied. Her tablets were crushed, suspended, and administered via the nasogastric tube. The Li dosage was increased to 1,000 mg/day. However, the Li concentration decreased to 0.43 mEq/L. Her manic symptoms slightly improved, and she agreed to take the medications by herself. Subsequently, the Li concentrations were 0.78 mEq/L on day 66 and 0.83 mEq/L on day 81. Progress and Li concentration was summarized in Figure 1.

The patient’s symptoms improved. However, some signs remained. Valproic acid 1,000 mg/day was added to her treatment regimen. On the 110th day of hospitalization, she was discharged. Finally, her symptoms improved.

**FIGURE 2:**
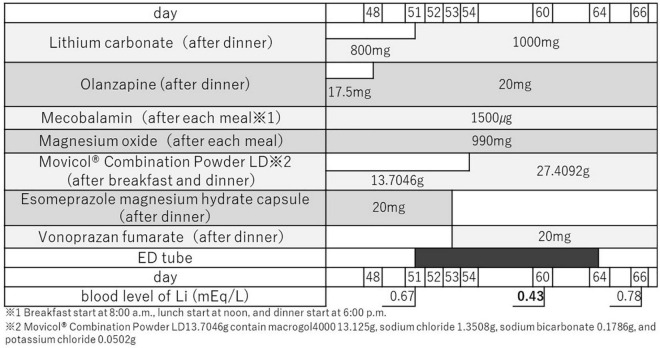
Each dosage of Li and concomitant drugs around the period of using the ED tube. During this period, the types of concomitant medications were not changed. ED tube: elemental diet tube.

The drug was administered once a daily at around 6:30 p.m. for renal protection, and the blood sample was drawn at around 12:30 a.m. on the next day. Our suspension method was as follows: Li and Vonoprazan Fumarate had been crushed by a crushing machine. Movicol^®^ Combination Powder LD is powder. Other drugs were crushed in mortar by hand using mortar sticks. We added crushed Li, Vonoprazan Fumarate, and Movicol^®^ Powder in the same vessel. Then we poured water (room temperature, soft water) about 20 ml and stirred them for a short time. Then we sucked them in a syringe, shook it once, then promptly put the suspension into the nasogastric tube. The tube was 8Fr. The length from the nose wing to the tip was 55 cm. Each dosage of Li and concomitant drugs around the period of using the elemental diet (ED) tube is shown in Figure 2. The blood tests performed before and after drug administration showed no evidence of dehydration. Yang mania Rating Scale (YMRS) score was 37/60 when she was admitted. It became worse 56/60 when physical restraint was needed. Then it improved to 1/60 when her symptoms were improved.

## Discussion

Herein, we report a patient who received Li via a nasogastric tube. The patient’s blood Li concentration decreased and then increased after the drug was administered orally, which improved manic symptoms. To the best of our knowledge, there are no reports about the use of Li administered via a nasogastric tube.

Li is one of the oldest drugs used to treat psychiatric disorders. It has antimanic, antidepressant, and mood-stabilizing effects and is a mood stabilizer used in treating bipolar disorder (2, 3). Further, it is a simple monovalent cationic alkali metal. Regarding its pharmacokinetics, absorption begins in the stomach, and 95–98% of the drug is absorbed in the small intestine. Almost 100% is absorbed, and the blood Li concentration peaks at approximately 2 h. The drug is commonly eliminated via the kidneys without being metabolized. The drug’s half-life is 15–18 h. It takes at least 7 days to reach a steady state (5). The Li concentration in the brain is about half of the serum concentration, and it is well correlated with the serum concentration (5). The side effect of renal dysfunction is less severe if it is administered once daily compared with divided doses twice daily (6). Li has a narrow effective therapeutic range. Therefore, to prevent Li poisoning, regular blood Li level monitoring and blood tests are necessary. Elderly patients with impaired renal function who are taking diuretics and who become dehydrated are more likely to develop poisoning symptoms. Therefore, food and fluid intake must be adequate, and the same Li dose should be taken regularly. The long-term use of oral Li increases the incidence of hypothyroidism and the risk of renal disorders such as interstitial nephritis (4). Hypercalcemia caused by hyperparathyroidism may occur (4). Li is an alkali metal and is a basic solution. The tablets are film-coated. Hence, they are easy to administer. In addition, the package insert states that “crushed and orally administered or crushed and orally administered via gavage is not recommended due to lack of data on its efficacy, safety, and pharmacokinetics.” If tablets are crushed, the drug is dissolved and absorbed more quickly, thereby increasing blood concentrations and the risk of poisoning (7).

In the current case, the medication was not crushed initially. Further, treatment was discontinued by surgeons during the perioperative period due to a high risk of intoxication caused by an unstable general medical condition. The patient’s condition worsened after treatment with Li was discontinued and she refused to take her medications. No other method except via the nasogastric tube can be used to administer Li. However, the blood Li level was assessed during administration, and it decreased unexpectedly. The following factors may have contributed to the following.

## Lesser amount of drug administered

The tablet was crushed and could have been lost during administration. According to Papiez et al., there was less amount of drugs lost when a pre-suspended solution was syringe-suspended than when a drug was directly placed into a syringe and administered (8). In the current case, drug loss could have occurred while making the pre-suspended solution. Further, Flanagan et al. showed that only the supernatant liquid might have been administered due to drug sedimentation during suspension (9). According to the Japanese package insert, Li carbonate does not dissolve easily in water. The components might have settled during suspension. Hence, the amount of drug administered could be lower.

## Changes in drug property

According to the Japanese package insert, Li carbonate is a strong basic suspension. Film-coated tablets contained several additives such as D-mannitol, corn starch, hydroxypropyl cellulose, hypromellose, magnesium stearate, and hardened oil. If they are crushed and suspended, the effects of these ingredients can disappear, and the property may differ from that of tablets.

## Drugs remaining in the tube

Crushed and suspended Li tablets could remain in the nasogastric tube, and this could decrease the amount of Li administered. Although the tube is flushed with water, a small amount of Li might remain in the tube.

## Lower absorption due to ionized form

Lithium carbonate suspension is a strongly basic solution. In addition, since carbonic acid is a divalent acid, there are two acid dissociation constant (pKa), 6.2 and 9.7, both of which values are higher than the pH of the stomach of 2.0. Therefore, when lithium carbonate suspension enters the stomach directly, ionized lithium increases. Lithium absorption may be reduced because the ionized form does not readily pass through cell membranes.

## Absorption speed

The peak blood Li concentrations could have shifted, and the blood concentrations might have been evaluated during the increase or decrease of blood concentrations. Moreover, the time of trough level could have shifted. According to Needham TE et al., seven commercially available Li carbonate products were administered in the crushed form to seven healthy adult male volunteers. Saliva and urine samples were collected hourly to measure concentrations and significant differences in peak concentrations in the saliva, which is attributed to rapid powder absorption (10). However, the interval time difference between the tablet and crushed tablet to trough level is still unknown.

## Low absorption

Fay et al. assessed the blood levels of levetiracetam administered using three methods (tablets with water, crushed with juice, and nutritional supplements). The area under the blood concentration-time curve (AUC) and the maximum drug concentration (Cmax) levels decreased, and the maximum drug concentration time (Tmax) was prolonged in the crushed form (11). In this case, the tablet was crushed and administered after dinner. Thus, crushing could have reduced absorption as the drug was taken with meals, thereby resulting in decreased blood concentrations.

## Strengths and limitations

This is a rare case in which the Li level of the patient was lower than expected due to administration route. This phenomenon could be attributed to the fact that the tablets were crushed. Therefore, several points should be considered if Li tablets are crushed and administered. Meanwhile, the blood Li concentration was measured only once when the drug was administered by grinding, which is not reproducible. To validate our result, the blood Li concentration should be evaluated several times.

## Take-away lessons from the case

Lithium carbonate administered via a nasogastric tube may be an option for patients who are in manic state and cannot take oral lithium carbonate. Nevertheless, caution should be observed due to the risk of fluctuations in blood lithium carbonate levels, as in the current case.

## Patient perspective

Caution has been taken to prevent the identification of the patient. Verbal and written informed consents were obtained from the individual involved in this case for the publication of any potentially identifiable images or data included in this article.

## Data availability statement

The raw data supporting the conclusions of this article will be made available by the authors, without undue reservation.

## Ethics statement

Ethical review and approval was not required for the study on human participants in accordance with the local legislation and institutional requirements. The patients/participants provided their written informed consent to participate in this study. Written informed consent was obtained from the individual involved in this case for the publication of any potentially identifiable images or data included in this article.

## Author contributions

RF treated clinically the patient of the case and wrote the case report. KF advised RF for the treatment of the patient. YO contributed to wrote the manuscript. All authors contributed to the article and approved the submitted version.
